# De novo exonic duplication of ATP1A2 in Italian patient with hemiplegic migraine: a case report

**DOI:** 10.1186/s10194-017-0770-x

**Published:** 2017-06-07

**Authors:** Stella Gagliardi, Gaetano Salvatore Grieco, Francesca Gualandi, Luisa Maria Caniatti, Elisabetta Groppo, Marialuisa Valente, Giuseppe Nappi, Marcella Neri, Cristina Cereda

**Affiliations:** 1 Genomic and Post-Genomic Center, “C. Mondino” National Neurological Institute, Mondino 2, 27100 Pavia, Italy; 2 Medical Genetics Unit, Department of Medical Sciences and Reproduction and Growth, University-Hospital S’Anna Ferrara, Ferrara, Italy; 3 Neurology Unit, Department of Neuroscience/Rehabilitation, University-Hospital S’Anna Ferrara, Ferrara, Italy; 4 Headache Science Center, C. Mondino National Neurological Institute, Pavia, Italy

**Keywords:** ATP1A2, Duplication, Hemiplegic migraine, De novo

## Abstract

**Background:**

Sporadic Hemiplegic Migraine is a rare form of migraine headache. Mutations in three different genes, two ion-channel genes and one encoding an ATP exchanger, *CACNA1A*, *ATP1A2* and *SCN1A* are all responsible for the FHM phenotype, thus indicating a genetic heterogeneity for this disorder. Here, we described a de novo exonic duplication of *ATP1A2* in an Italian patient with Hemiplegic Migraine.

**Case presentation:**

We describe the case of a young woman (33 year old) who suffered from the age of 8 years of episodic weakness of the limbs, associated to other subjective and objective features. From aged 25, she developed neurological symptoms, like dizziness, blurred vision and an MRI scan revealed aspecific peritrigonal white matter hyperintensities. Aged 32 she suffered of right hemisomatic sudden-onset paresthesias, hypoesthesia and hyposthenia and the patient was genetically investigated for sporadic hemiplegic migraine.

**Conclusions:**

Here we report, for the first time, an exonic duplication in the *ATP1A2* associated with hemiplegic migraine. The variation identified involves exon 21 of the *ATP1A2* and is expected to alter the function of the alpha(2) subunit of the Na(+)/K(+) pump; the de novo nature of the duplication further supports its pathogenic role. To date, no other CNVs have been described in the *ATP1A2* but only point mutations are reported. The novel mutation may result impaired M9 transmembrane domain, in a loss-of-function of the alpha(2) Na(+)/K(+)-ATPase with glutamate accumulation, alteration of synaptic function and neurotransmission.

## Background

Hemiplegic migraine (HM) is a rare form of migraine headache. There are two types of hemiplegic migraine–familial hemiplegic migraine (FHM) and sporadic hemiplegic migraine (SHM). Migraines typically cause intense, throbbing pain in one area of the head. Some people with migraines also experience nausea, vomiting, and sensitivity to light and sound [1, 2]. These recurrent headaches typically begin in childhood or adolescence and can be triggered by specific foods, emotional stress, and minor head trauma. Each headache may last from a few hours to a few days. Mutations in three different genes, two ion-channel genes and one encoding an ATP exchanger, calcium voltage-gated channel subunit alpha1 A (*CACNA1A),* ATPase Na+/K+ transporting subunit alpha 2 *(ATP1A2)*, Sodium channel protein type 1 subunit alpha (*SCN1A)* are all responsible for the HM phenotype, thus indicating a genetic heterogeneity for this disorder [3, 4]. Recently, also proline rich transmembrane protein 2 (*PRRT2)* has been associated to HM [4]*. ATP1A2* is located on 1q23 (FHM2, MIM #182340) [5], and more than 60 mutations have been identified in association to the FHM phenotype. This gene, *ATP1A2*, encodes the alpha-2 catalytic subunit of a sodium-potassium-ATPases [5]. Mutations in *ATP1A2* are mainly missense; few deletions have been described [5] but so far no duplications in *ATP1A2* have been discovered [3]. In this study, we describe, for the first time, a novel ATP1A2 exon duplication in an Italian patient with SHM phenotype.

## Case presentation

We describe the case of a young woman who suffered from the age of 8 years of episodic weakness of the limbs, associated to other subjective and objective features.

The onset was with recurrent episodic fatigue, limb weakness with dysesthesias, paresthesias, hyperalgesias, pain triggered from febrile illness, arthralgias in all four limbs; furthermore she reported episodes of skin rash. The painful symptoms were categorized as a fibromyalgia and she underwent amitriptyline and ademetionine therapy without relief. Until the age of 25 years she was taken into care by rheumatologists and, given the concomitant presence of stomatitis, The Raynaud phenomenon, and episcleritis, a form of Behcet disease (HLA-B51 negative) was diagnosed, then disconfirmed, but meanwhile treated with steroids, azathioprine, colchicine. From year 2007, aged 25, she developed neurological symptoms, like dizziness, blurred vision and an MRI scan revealed aspecific peritrigonal white matter hyperintensities.

**Fig. 1:**
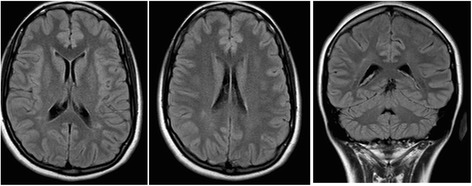
MRI scan images revealing aspecific peritrigonal white matter hyperintensities. The control MRI was performed after 6 months and 1 year and confirmed the small focal peritrigonal white matter hyperintensities in T2/FLAIR. Hypertension, heart valvular disease and other vasculophaties were excluded. Transcranial doppler US no detected the micro-embolic signals. Another MRI was performed after 2 years and the picture is unvaried

Aged 32 she suffered of right hemisomatic sudden-onset paresthesias, hypoesthesia and hyposthenia, confirmed on examination; this episode lasted 45 min and it was followed by headache, sleepness, diplopia. She was admitted on ED: a CT scan was negative; a mild antinuclear antibodies positivity (1:160) was found andan hemato-encephalic-barrier dysfunction on liquoral analysis, liquoral analysis showed an hemato-encephalic-barrier dysfunction with increased protein amount (57 mg/dl) without intratecal synthesis of IgG. CSF/serum albumin ratio (QAlb) = 9.43. A new MRI scan confirmed the previous result (Fig. 1). There were no abnormalities at the neurophysiological evaluation. The ophthalmologic evaluation revealed a bilateral hypovisus (7–8/10 in the right and 4–5/10 in the left), lax eyelid syndrome, bilateral posterior lenticonus, astigmatism, exothropia, monocular bilateral diplopia; OCT scan was normal. During hospitalization the patient suffered from episodic intense headache, with migrainous features.

The following year she suffered from another episode of right hemiparesis and hemi-hypoesthesia associated to sub-continuous diffuse arthromyalgia. After assumption of a tablet of triptane, she referred a severe crisis and between the crisis of migraine with motor aurea muscle pain and fatigue were present.

As the persistent headache, the patient started prophylactic long-term therapy with topiramate, with beneficial effects.

**Fig. 2:**
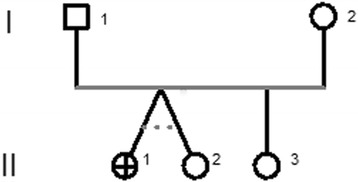
Pedigree of the Italian family. Filled symbols represent affected individuals and open symbols non-affected individuals

Due to the concomitant presence of recurrent hemiparesis associated or subsequent to migraine attacks, based on the headache classification criteria [6], the patient was genetically investigated for familial hemiplegic migraine (FHM); the familial history was negative for neurological disorders (Fig. 2), both parents and the twin sister never experienced migraine attack similar to the one of our patient.

### Molecular analysis

DNA was extracted from peripheral blood cells using standard procedures. *ATP1A2* and *CACNA1A* exons were amplified by PCR using primers located in adjacent intronic regions. The amplicons were screened for sequence variations by direct sequencing using the Big-Dye Terminator v3.1 sequencing kit (Applied Biosystems, Milan, Italy) and ABI 3130 Genetic Analyser (Applied Biosystems, Milan, Italy). The alignment to reference sequence was performed using Sequencher 4.8 software. Primer sequences and PCR conditions are available upon request.

Search for CNVs was performed using MLPA assay using both the P348-A2 ATP1A2-CACNA1A probe mix and P279 CACNA1A probemix (SALSA MLPA Kit, MRC-Holland). Moreover also the second kit about migraine has been used for each sample (SALSA MLPA; MRC-Holland).

Identified *ATP1A2* duplication was confirmed by Real Time PCR. All the experiments have been performed in triplicates.

## Conclusion

**Fig. 3:**
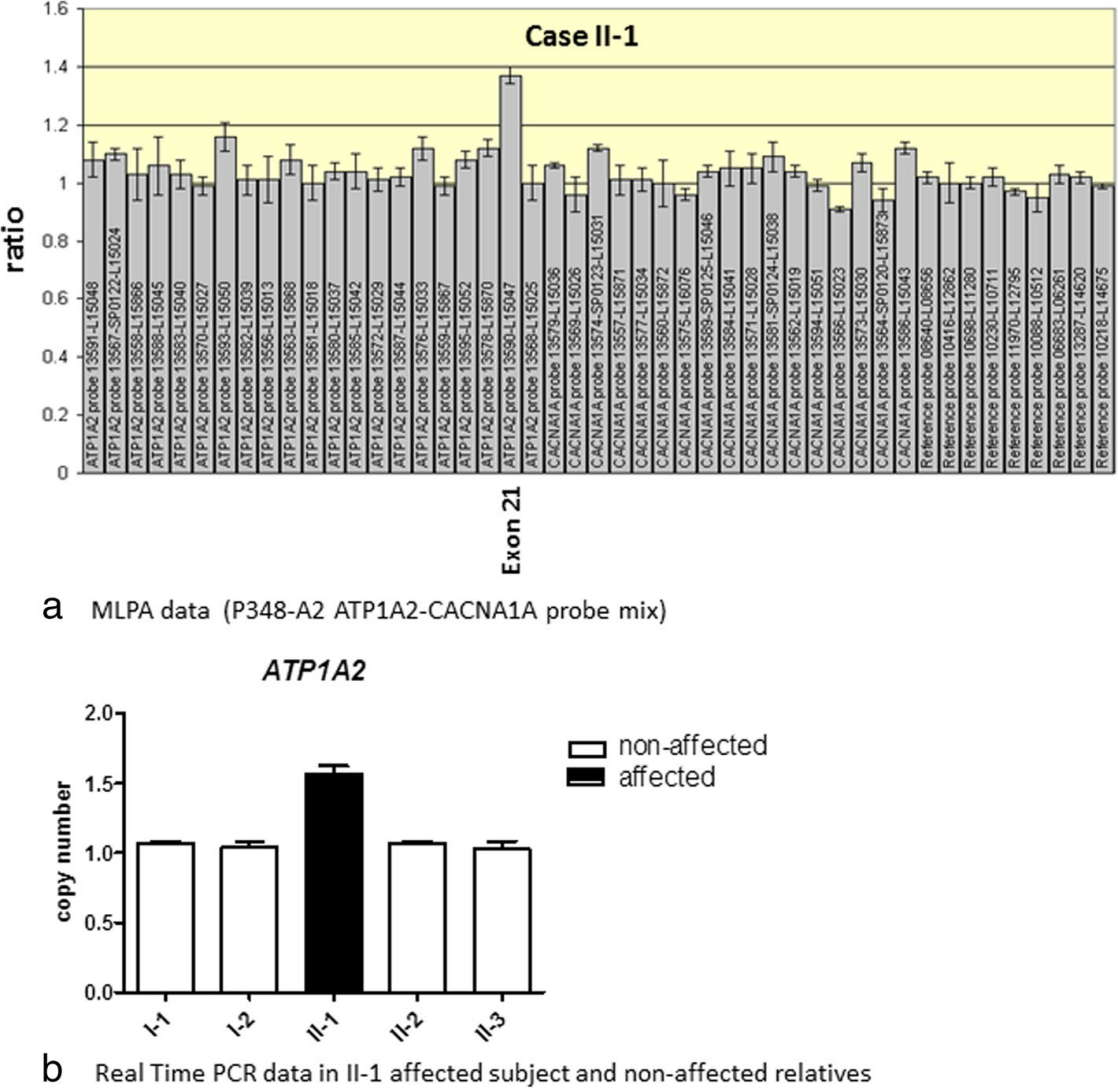
**a** ATP1A2 duplication in SHM patient obtained by MLPA assay. For each probe, the ratio < 0.75 stands for deletion; and the ratio > 1.3 stands for duplication. **b** ATP1A2 quantification by Real Time PCR in patient and in unaffected relatives

We report here for the first time the identification of an exonic duplication in the *ATP1A2* associated with hemiplegic migraine. MLPA test identified a duplication involving the exon 21 of *ATP1A2* (plus 50% amplification in the signal) with multiple MLPA probes (Fig. 3a). ATP1A2 exonic duplication was not detected in 100 healthy subjects and 100 FHM patients. The identified exon 21 duplication resulted de novo at the segregation analysis in both patient’s parents. Direct sequencing analysis was negative for small mutations both in *CACNA1A* and in *ATP1A2* exons**.**

Mutations of the genes encoding the Na/K-ATPase a-subunit (ATP1A2 and ATP1A3) have been described in three distinct human neurological disorders: familial hemiplegic migraine, rapid onset dystonia Parkinsonism, and alternating hemiplegia of childhood [3]. These neurological disorders are dominantly inherited and caused primarily by missense mutations, which are thought to confer a mild hypomorphic loss-of-function.

In the first two families in which FHM was associated to mutations in *ATP1A2* [7], six several affected individuals presented a history of seizures independent of hemiplegic migraine attacks; the penetrance of epilepsy is also incomplete in these families.

The clinical presentation in our patient is peculiar due to the fact that she experienced since childhood recurrent weakness with dysesthesias/paresthesias of limbs and pain triggered by fever, diagnosed firstly as fibromyalgia. From the age of 25, she developed more neurological symptoms, like dizziness, blurred vision and she suffered of hemisomatic sudden-onset paresthesias/hyposthenia, associated to severe headache. After assumption of a tablet of triptane, she referred a severe crisis and between the crisis of migraine with motor aurea muscle pain and fatigue were present.

Although the mutations in *ATP1A2* that cause hemiplegic migraine are not as well-studied, it is thought that they may impair the transport of sodium and potassium ions and prolonging the presence of neurotransmitters between neurons. The abnormal signalling resulting from these changes leads to the headaches and auras characteristic of the condition.

In fact, starting from hemiplegic migraine clinical features, these patients suffer from migraines with hemiplegia and partial paralysis during the aura phase and, in some cases, accompanied by seizures or cognitive dysfunction. Neuroimaging studies have shown that migraine aura is caused by cortical spreading depression (CSD) [8]. Inhibition of ATP1A2 leads to high levels of extracellular potassium, causing neurons to become depolarized which can cause CSD [9]. The penetrance of hemiplegic migraine is incomplete (about 65%), and mutation carriers may be affected by typical migraine with aura but not by hemi-plegic migraine [7].

To our knowledge, this report is the first to describe an exonic copy number change in *ATP1A2* associated to a clinical phenotype of hemiplegic migraine.

Several data support the possible pathogenic role of this variation; firstly the de novo nature of the variation itself, not inherited forms the healthy parents. Secondly, this copy number variation as not identified in 100 healthy controls neither in 100 hemiplegic migraine patients. Moreover, our hypothesis is supported by the guideline for variant interpretation of copy number variation [10] that suggests that if CNV is shown to represent a de novo mutation in the proband, this is generally taken as evidence supporting pathogenicity.

So far, the mechanism for the effects of ATP1A2 mutations is not clear. Moreover, point mutations in exon 21 or in flanking exons are reported as pathogenic in HM patients. These mutations result in a loss-of-function of the alpha(2) Na(+)/K(+)-ATPase with glutamate accumulation, alteration of synaptic function and neurotransmission. Also the duplication of exon 21 of *ATP1A2* is expected to alter the function of the alpha(2) subunit of the Na(+)/K(+) pump by the impairment of domain M9, where exon 21 is located.

The hypothesized mechanism for the effects of ATP1A2 duplication is that may cause an increase in extracellular potassium and intracellular sodium which increases intracellular calcium levels through the Na+/Ca2+ exchanger, resulting in glutamate release and a decrease in glutamate clearance which can also lead to CSD. This hypothesis results in making the brain more susceptible to CSD and therefore migraines with aura [11].

In conclusion, this work further strengthens the involvement of the *ATP1A2* in hemiplegic migraine and highlights the importance to complement analysis of direct sequencing with quantitative gene analysis to exactly describe the association between ATP1A2 variations and hemiplegic migraine.

## Key findings bullet points

- Identification of novel mutation in ATP1A2 gene associated to Sporadic Hemiplegic Migraine
- Discovery of a duplication of exon 21 of ATP1A2 gene
- highlights the importance to complement analysis of direct sequencing with quantitative gene analysis
