# Erratum to: De novo exonic duplication of ATP1A2 in Italian patient with hemiplegic migraine: a case report

**DOI:** 10.1186/s10194-017-0771-9

**Published:** 2017-07-12

**Authors:** Stella Gagliardi, Gaetano Salvatore Grieco, Francesca Gualandi, Luisa Maria Caniatti, Elisabetta Groppo, Marialuisa Valente, Nappi Giuseppe, Marcella Neri, Cristina Cereda

**Affiliations:** 1 Genomic and Post-Genomic Center, “C. Mondino” National Neurological Institute, Mondino 2, 27100 Pavia, Italy; 2 Medical Genetics Unit, Department of Medical Sciences and Reproduction and Growth, University-Hospital S’Anna Ferrara, Ferrara, Italy; 3 Neurology Unit, Department of Neuroscience/ Rehabilitation, University-Hospital S’Anna Ferrara, Ferrara, Italy; 4 Headache Science Center, C. Mondino National Neurological Institute, Pavia, Italy

## Erratum

In the original publication [1] was the name of one author in incorrect order.

Wrong: Nappi Giuseppe

Correct: Giuseppe Nappi

The original article was updated to rectify this error.
